# Frequency of HLA‐DQ, susceptibility genotypes for celiac disease, in Brazilian newborns

**DOI:** 10.1002/mgg3.444

**Published:** 2018-07-16

**Authors:** Fernanda C. Almeida, Lenora Gandolfi, Karina N. Costa, Marilucia R. A. Picanço, Lucas M. Almeida, Yanna K. M. Nóbrega, Riccardo Pratesi, Claudia B. Pratesi, Nicole Selleski

**Affiliations:** ^1^ Graduate Program in Medical Sciences School of Medicine University of Brasilia Brasilia DF Brazil; ^2^ Research Center for Celiac Disease School of Medicine University of Brasilia Brasilia DF Brazil; ^3^ Graduate Program in Health Sciences School of Health Sciences University of Brasilia Brasilia DF Brazil; ^4^ Department of Pediatrics School of Medicine University of Brasilia Brasilia DF Brazil; ^5^ Department of Pharmaceutical Sciences School of Health Sciences University of Brasilia Brasilia DF Brazil

**Keywords:** celiac disease, frequency, HLA‐DQ2, HLA‐DQ8, newborn, polymerase chain reaction

## Abstract

**Background:**

The frequency of HLA‐DQ2 and DQ8 predisposing genotypes for celiac disease (CD) has shown significant variation among different world regions and has not been previously determined among the highly interbred Brazilian population. The aim of this study was to investigate the frequency of these genotypes among Brazilian newborns (NB).

**Methods:**

We typed DQA1*05 ‐ DQB1*02 (DQ2.5) and DQA1*03 ‐ DQB1*03:02 (DQ8) alleles in 329 NB using qPCR technique. Subsequently we confirmed our results by PCR‐SSP using a reference kit which further identified DQ2.2 (DQA1*02:01 ‐ DQB1*02).

**Results:**

Among the 329 NB, using qPCR technique: 5 (1.52%) carried both DQ2.5 and DQ8 variants; 58 (17.63%) carried only DQ2.5 (DQA1*05 and DQB1*02) and 47 (14.29%) carried only the DQ8 (DQA1*03 and DQB1*03:02) variant. The use of the PCR‐SSP method yielded further information; among the 329 samples: 34 (10.34%) tested positive for DQ2.2 and among the 47 previously DQ8 positives samples, we found 10 (3.04%) that also tested positives for DQ2.2.

**Conclusion:**

43.7% of the analyzed individual tested positive for at least one of the CD predisposing HLA‐DQ genotypes in our group of Brazilian NB. The highest frequency was found for DQ2.5 positive subjects (17.6%) followed by DQ8 (11.3%); DQ2.2 (10.3%); DQ8 and DQ2.2 (3.0%); DQ2.5 and DQ8 (1.5%). We found no positive sample for DQ2.5 associated with DQ2.2.

## 1 INTRODUCTION

Celiac disease (CD) is an immune‐mediated inflammatory disease of the small intestine that is mainly triggered and maintained by the storage proteins (gluten) of wheat, barley, and rye in genetically predisposed individuals (Schuppan, Junker, & Barisani, 2009). Accurate epidemiologic studies have disclosed that CD affects approximately 1% of the general population, both in Europe and North America (Catassi et al., 2007; Mustalahti et al., 2010). The prevalence of CD in developing countries has shown significant variations, being rare in some and very common in others (Cataldo & Montalto, 2007) most likely due to differences in frequency of specific alleles that code for HLA class II molecules (Abadie, Sollid, Barreiro, & Jabri, 2011). Screening studies in Brazil have shown variable results when analyzing CD prevalence rates among different regions of the country, ranging from 1:119 to 1:417 in the general population (Crovella et al., 2007; Pratesi et al., 2003) and 1:214 to 1:681 in presumably healthy blood donors (Gandolfi et al., 2000; Oliveira et al., 2007).

Genetic susceptibility to CD is associated with two sets of HLA‐DQA1 and DQB1 alleles (MIM: 146880 and 604305, respectively); DQA1*05 ‐ DQB1*02 and DQA1*03 ‐ DQB1*03:02, which code for class II MHC DQ2.5 and DQ8 molecules respectively (Sollid, Gunnar Markussen, Gjerde, Vartdal, & Thorsby, 1989). HLA‐DQ2.5 is either expressed in *cis* (encoded by HLA‐DR3‐DQA1*05:01‐DQB1*02:01) or *trans* configuration, encoded by HLA‐DR11‐DQA1*05:05‐DQB1*03:01 (HLA‐DQ7.5), and HLA‐DR7‐DQA1*02:01‐DQB1*02:02 (HLA‐DQ2.2) (Mubarak et al., 2013). Together HLA‐DQA1*05:05 and DQB1*02:02 code for a heterodimer highly similar to HLA‐DQA1*05:01 ‐DQB1*02:01 and therefore it could be also called HLA‐DQ2.5 (Mubarak et al., 2013). Approximately 90%–95% of celiac patients are HLA‐DQ2.5 positive, and half of the remaining patients are HLA‐DQ8 positive (Karell et al., 2003; Louka & Sollid, 2003). According to recent studies the most prevalent haplotype found in patients lacking HLA‐DQ2.5 and HLA‐DQ8 is HLA‐DQ2.2 (Mubarak et al., 2013). However, previous studies performed in a smaller Brazilian population sample suggest that HLA‐DQ2.2 can only be considered a risk factor when associated with DQ2.5 (Almeida et al., 2016; Selleski et al., 2018).These variants are responsible for only 40% of the genetic risk of CD and are carried by approximately 30% of the general Caucasian population (Megiorni & Pizzuti, 2012), thus suggesting that HLA is only one of the regions that could confer risk of developing this condition. However, the other 60% of the genetic susceptibility to CD is shared by HLA class I and non‐HLA genes, each of which is estimated to contribute only with a small risk effect (Trynka et al., 2011; Withoff, Li, Jonkers, & Wijmenga, 2016).

The frequency of DQ2.5 and DQ8 genotypes has shown significant variations among different populations (Catassi & Yachha, 2008). Previous studies performed in the Brazilian population showed a significant genetic heterogeneity. Confirming the genetic heterogeneity of the Brazilian population using an analysis of ancestry informative markers from individuals derived by birth from the five geopolitical Brazilian regions, Lins, Vieira, Abreu, Grattapaglia, and Pereira (2010) disclosed a major contribution of European ancestry (0.771) followed by African (0.143) and Amerindian (0.085) ancestries. A screening to determine the frequency of CD predisposing alleles in a representative sample of a highly admixed population as the Brazilian population has not been previously performed. Consequently, the aim of the present study was to determine the frequency of CD HLA‐DQ predisposing genotypes in a representative group of newborns (NB) from the city of Brasilia, Brazil.

## 2 MATERIAL AND METHODS

### 2.1 Ethical compliance

The study complied with the principles of the latest Declaration of Helsinki (2008) and was approved by the Research Ethics Committee on Medical Science of the University of Brasilia, School of Medicine (protocol No 132/2008).

### 2.2 Study population

Mothers‐to‐be admitted to the maternity ward of the Brasilia University Hospital (HUB) during February 2012 and March 2013 received extensive exposition of the research objectives and provided written consent for the participation of their forthcoming NB in the study protocol. HUB is a public hospital that belongs to the Brazilian Unified Health System and focuses on care of low‐income populations located in different neighborhoods and in the outskirts of Brasilia. All NB were eligible for the screening, despite the ones with low birth weight, prematurity, or severe congenital abnormalities at the time of birth. Our study population consisted in a total of 329 NB, 143 were males and 186 females. Immediately after birth, 10 ml of umbilical cord blood (UCB) was collected: 5 ml were used for routine tests the other 5 ml were collected in sterile EDTA‐coated tube for DNA extraction. This last procedure was performed within the first 48 hr using Illustra™ Blood genomic Prep Mini Spin kit (Healthcare, Buckinghamshire, UK) according to the manufacturer's instructions. Concentration of DNA samples were adjusted to 15 ng/μg after being quantified at 260 nm using Nanodrop ND‐1000 Spectrophotometer (Nanodrop Technologies, Wilmington, DE, USA).

Collection of UCB for screening of HLA markers in NB has been successfully used in research studies, including large screening programs, to determine the genetic risk for diabetes type 1 (Berzina, Shtauvere‐Brameus, Ludvigsson, & Sanjeevi, 2002; Emery et al., 2005). Using an appropriate collection technique, the chance of maternal blood contamination is negligible (Rewers et al., 1996).

### 2.3 qPCR HLA‐DQ typing

DQA1*05 ‐ DQB1*02 (DQ2.5) and DQA1*03 ‐ DQB1*03:02 (DQ8) amplifications were performed, separately, using qPCR technique (Step One Real Time PCR System; Applied Biosystems, Life Technologies™, Carlsbad, CA, USA). Hybridization was accomplished using sequence‐specific primers for DQA1*05, DQB1*02 and DQA1*03 as described by Olerup and Aldener (1993), and for DQB1*03:02 as described by Profaizer, Eckels, and Delgado (2011). Primers for human growth hormone were used as an internal control (Profaizer et al., 2011). Each primer was used at a final concentration of 0.5 μM.

Amplifications of DQA1*05, DQB1*02 and DQA1*03 were performed following a qPCR protocol developed in our laboratory (Selleski et al., 2015). Typing of the DQB1*03:02 allele was performed as described by Profaizer et al. (2011).

### 2.4 PCR‐SSP HLA‐DQ typing

All positive samples obtained on the previous qPCR typing underwent further testing using DQ‐CD TypingKitPlus (BioDiagene, Palermo, Italy) according to the manufacturer's recommendations. The DQ2.5 heterodimer was identified by the presence of the alleles DQA1*05 and DQB1*02, and the DQ8 heterodimer was identified by the presence of DQA1*03 and DQB1*03:02. This method allowed us to identify DQ2.5 alleles in both cis and trans configuration. Furthermore, the kit contains primers for the identification of DQB1*02 allele homozygosis and other CD associated genotypes like DQ2.2.

### 2.5 Statistical methods

Data analysis was performed using the Statistical Package for Social Sciences (SPSS Statistics for Windows, Version 17.0; SPSS Inc, Chicago , IL, USA).

## 3 RESULTS

From a total of 329 NB (143 males and 186 females), 187 (56.84%) (77 males and 110 females) were positive for at least one HLA predisposing allele in qPCR assays. Analysis of the results showed 5 (1.52%) NB carrying both DQ2.5 and DQ8 genotypes, 58 (17.63%) were positive only for DQ2.5 (DQA1*05 and DQB1*02), and 47 (14.29%) were positive for DQ8 only (DQA1*03 and DQB1*03:02), 10 (3.04%) of them also showed the DQB1*02 allele. From the samples that only tested positive for one of the searched alleles, 34 (10.33%) were positive for DQB1*02 and 43 (13.07%) were positive for DQA1*05. It was not possible to determine the genotype conformation on these last samples using qPCR. Results obtained by qPCR were confirmed by the PCR‐SSP method which, in addition to the identification of the four previously tested alleles, was able to detect DQB1*02 homozygosis and allowed the differentiation of other allele combinations e.g. DQ2.2 (Table 1).

**Table 1:**
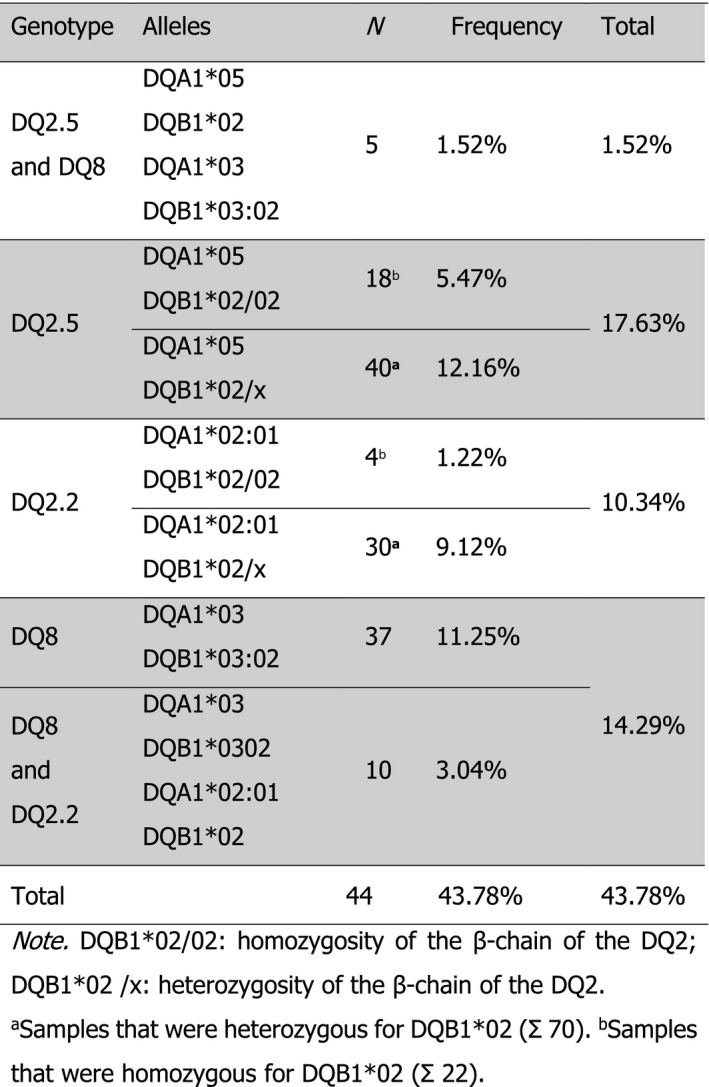
Frequency of CD predisposing HLA‐DQ genotypes in the 144 positive samples using PCR‐SSP

## 4 DISCUSSION

We performed the current study using UCB obtained at the time of birth from a random sample of NB. This population‐based design avoids the exclusion of CD subject as part of the general population, which is a common bias in this kind of studies (Alarida, Harown, Di Pierro, Drago, & Catassi, 2010; Megiorni & Pizzuti, 2012). Besides, other types of study designs do not consider the probable loss of individuals due to early mortality rate, a matter that still concerns physicians of developing countries (Pratesi et al., 2003).

In the current study, the frequency of HLA‐DQ2 and/or DQ8 variants was 43.7%, which can be considered similar to the results obtained in the European population, in which different studies showed a frequency between 30%–50% (Husby et al., 2012). Several studies identified DQ2.2 as a predisposing genotype for CD, they also showed that this variant is even more prevalent, between celiac patients, than DQ8 (Fernandez‐Arquero, Figueredo, Maluenda, & de la Concha, 1995; Karell et al., 2003; Mubarak et al., 2013; Polvi et al., 1998). Even though this kind of molecule does not bind as efficient as DQ2.5 to gluten peptides, it does strong enough to build up a T‐cell antigluten response, leading to the development of CD (Bodd, Kim, Lundin, & Sollid, 2012). A previous study performed by our group confirmed the relevance of this genetic variant in the Brazilian population (Almeida et al., 2016).

When comparing our population with another Latin‐American country as Mexico, we found a slightly increased frequency of CD HLA‐DQ predisposing variants in our population (43.7% vs. 28.8%). However, authors did not consider DQ2.2 in the Mexican study (Mejía‐León, Ruiz‐Dyck, & Calderón de la Barca, 2015). An unexpectedly high prevalence of this HLA‐DQ genotypes (55.9%) was reported in a recent study from Australia. They observed an increased frequency of both DQ2 variants (DQ2.5: 24.5%; DQ2.2: 15.5%) (Anderson et al., 2013). We found similarities between our results and that from a Latvian study when comparing the total predisposing variants (43.7% vs. 41.2%), although they have reported a higher frequency of DQ2.5 and lower number of DQ8 positive individual than in our population sample (Leja et al., 2015). Our study was also comparable with a research performed in the Danish population, which analyzed the same genetic variants as assessed in our survey (Table 2). However, they found a higher number of DQ2.5 positive samples (Karhus, Thuesen, Skaaby, Rumessen, & Linneberg, 2018).

**Table 2:** Frequency of CD (HLA‐DQ) predisposing variants in general population from different countries

	Brazil (%)	Denmark^a^ (%)
DQ2.5 and DQ8	1.5	2.5
DQ2.5	17.6	22.2
DQ2.2	10.3	7.3
DQ8	11.3	12.0
DQ8 and DQ2.2	3.0	1.2
DQ2.5 and DQ2.2	0	1.2
Total	43.7	46.4

a (Karhus et al., 2018).

This difference in the frequency of specific genetic variants, could be explained by the high degree of heterogeneity of the Brazilian population, resulting from five centuries of interbreeding among people of three main ethnicities: Amerindians, Europeans, and Africans. In addition to this initial ethnic contribution, during the last two centuries, successive migratory waves, mainly comprising Italians, Spaniards, Germans, Japanese, Lebanese, and Syrians, further increased the racial diversity of the population. Consequently, each Brazilian has a singular proportion of these ethnic origins, which hampers his/her precise characterization in a specific racial group (Pena, Bastos‐Rodrigues, Pimenta, & Bydlowski, 2009). The current population of Brasilia can be considered fairly representative of the Brazilian population since, during more than 50 years from its foundation, this city, with more than 2,500,000 inhabitants (IBGE – Instituto Brasileiro de Geografia e Estatística, 2010), has hosted people from all over the country.

The high frequency of predisposing genotypes in our population was an unexpected result as several screening studies performed in different regions of Brazil had already shown a lower CD frequency, when compared with the one found in Europe (Crovella et al., 2007; Gandolfi et al., 2000; Oliveira et al., 2007). We suggest that this discrepancy between the presence of an increased genetic risk and a lower frequency of CD in our population could be explained by an increased mortality due to undiagnosed CD among the younger groups. Previous studies performed by our group seem to support this possibility. In a prior screening study developed in the same region, we found an unexplained variation on CD frequency among different age groups, with an increased number of cases in the younger group (Pratesi et al., 2003). Additionally, we could not find any case of CD in 946 elderlies (Almeida et al., 2013) contrasting with the results obtained by Vilppula et al. (2009) in Finland that showed an increased prevalence of CD among individuals over 52 years.

This study possessed limitations. Although contamination of NB samples by maternal blood did not appear to be a significant problem, its exact frequency should be determined in the future by retyping the HLA‐DQ2 and DQ8 genotypes from blood samples obtained directly from the child (Rewers et al., 1996). Additionally, although the population of Brasilia can be considered a good representation of the Brazilian population, this is only partially true because the contribution of people coming from the Midwestern and Northeastern regions of Brazil in which the influence of Afro‐descendants and Amerindians prevail is different when compared to people from the Southern states of Brazil (Godinho et al., 2008; Lins et al., 2010).

In conclusion, 43.7% of the analyzed individual tested positive for at least one of the CD predisposing HLA‐DQ genotypes in our group of Brazilian NB. The highest frequency was found for DQ2.5 positive subjects (17.6%) followed by DQ8 (11.3%); DQ2.2 (10.3%); DQ8 and DQ2.2 (3.0%); DQ2.5 and DQ8 (1.5%). We found no positive sample for DQ2.5 associated with DQ2.2.

## CONFLICT OF INTEREST

The authors declare no conflict of interest.
